# Effective communication in eliciting and responding to suicidal thoughts: a systematic review protocol

**DOI:** 10.1186/s13643-016-0211-y

**Published:** 2016-02-17

**Authors:** Rose McCabe, Ruth Garside, Amy Backhouse, Penny Xanthopoulou

**Affiliations:** College House, University of Exeter Medical School, St Luke’s Campus, Heavitree Road, Exeter, EX1 2LU UK; European Centre for Environment and Human Health, Knowledge Spa, Royal Cornwall Hospital, Truro, TR1 3HD UK

**Keywords:** Suicide, Suicidal ideation, Systematic review, Protocol, Controlled studies, Effective communication

## Abstract

**Background:**

In the UK, over 6500 people die by suicide each year. In England alone, this is one person every 2 h. Professionals assess risk of suicide in face-to-face contacts with people potentially at risk. The National Confidential Inquiry into Suicide found that most people who took their life were classified as ‘low risk’ in their final contact with mental health services. Training for front-line staff in reducing suicide is a NHS priority. While there is considerable evidence on *what* to assess when exploring suicidal ideation, there is little evidence on *how* to ask sensitive questions to effectively identify suicide risk and how to respond in the treatment encounter to reduce patient distress and suicidal ideation. This is critical for identifying risk and putting appropriate care in place.

**Methods:**

An electronic search will be conducted using MEDLINE, CINAHL, Cochrane Library, EMBASE and PsycINFO databases. Controlled studies of effectiveness will be identified using a predefined search strategy. The focus will be on suicidal thoughts/feelings rather than self-harm without intent to die. Two authors will independently screen articles using predefined inclusion and exclusion criteria and relevant data will be extracted using the Cochrane Collaboration data extraction form for randomised controlled trials (RCTs). Discrepancies between the two authors will be resolved by consensus or by consulting a third author at all levels of screening. We will assess the quality of evidence as well as risk of bias. A meta-analysis will be conducted if participants, interventions and comparisons are sufficiently similar, and we will perform the meta-analysis using Stata data analysis and statistical software.

**Discussion:**

The results of this systematic review will be used to guide training and practice for health care professionals.

**Systematic review registration:**

PROSPERO CRD42015025867

**Electronic supplementary material:**

The online version of this article (doi:10.1186/s13643-016-0211-y) contains supplementary material, which is available to authorized users.

## Background

### Rationale

Every 2 h in England, someone takes their own life. Every year, around 170,000 people make an attempt on their life. The National Confidential Inquiry into Suicide [1] found that most people who took their life were classified as ‘low risk’ in their final contact with mental health services. The cross-government strategy ‘Preventing Suicide in England’ highlights the role of front-line staff training in reducing suicide [2]. Currently, training for healthcare professionals is not based on evidence about how to communicate to elicit and respond to suicidal ideation. Although there are standardised research interviews on *what* to assess when exploring suicidal ideation, there are no widely recognised clinical standards for *how* to ask sensitive questions in the treatment encounter and how to respond to reduce patient distress and suicidal ideation. This is critical for identifying risk, putting appropriate care in place and reducing suicidal ideation.

Shea [3] has developed the CASE approach, a guide for interviewing patients to elicit suicidal ideation, which emphasises the importance of asking the right questions. Gask et al. [4] developed the widely used STORM training package. Other training studies have also improved professionals’ self-rated competence [5]. A recent study by de Beurs et al. [6] trained mental health teams in applying suicide guidelines and found a positive effect on professional self-rated competence and guideline adherence assessed by responding to online video clips. However, there is a dearth of research on actual professional-patient communication in the clinical encounter when assessing suicidal thoughts.

Communicating about suicide is complex, and it will not be possible to prevent all suicides. Nonetheless, there is an urgent need to identify empirical evidence for effective communication.

The aim of this review is to identify which communication strategies have been researched and which are effective in eliciting and responding to suicidal thoughts in institutional settings. We developed the protocol for the review in accordance with the PRISMA-P (2015) statement [7] for preferred reporting items for systematic review and meta-analysis protocols (see Additional file 1).

### Objectives

The aim of this review is to identify communicative techniques to elicit and respond to suicidal thoughts, and to assess their effectiveness in identifying suicide risk and reducing patient distress, suicidal ideation, suicide attempts and self-harm. The review question will be addressed through two objectives:

1. To describe these communication techniques.
2. To identify controlled studies that assess the effectiveness of communication strategies in eliciting and responding^1^ to suicidal ideation, i.e. suicidal thoughts and feelings.

## Methods/design

### Eligibility criteria

This systematic review will include published controlled studies that have reported results of communication techniques that are effective in eliciting and responding to suicidal thoughts in institutional settings. Criteria for excluding studies are presented below.

#### Types of studies

No restrictions will be placed on study location or publication date of included studies. We will include cluster randomised controlled trials, randomised controlled trials, controlled before-and-after studies and controlled pre-test/post-test designs. We will exclude non-controlled studies.

#### Types of participants

We will include interactions between participants of any age and gender who are potentially at risk of suicide and *the* professionals/paraprofessionals (e.g. lay mental health workers, nursing assistants, educators, volunteers) assessing them.

#### Types of intervention

We will include studies of interventions that are delivered in any setting to the specified population. We will include studies focusing on:

- Professional/paraprofessional-patient interaction
- Eliciting and responding to suicidal thoughts and feelings
- Communication between at least one professional/paraprofessional and one patient; other people can be present
- Institutional encounters
- Focus on suicidal ideation rather than diagnostic conditions, e.g. depression, anxiety, borderline personality disorder
- Interactions that take place within institutional settings or in the community

We will exclude studies that involve the following:

- Assisted suicide
- Self-harm without intent to die, defined as: non-suicidal self-injury: the direct, deliberate destruction of one’s own body tissue in the absence of intent to die. It differs from suicide attempt with respect to intent, lethality, chronicity, methods, cognitions, reactions, aftermath, demographics and prevalence [8]
- Psychological therapy without a specific focus on eliciting and responding to suicidal ideation (e.g. Dialectical Behaviour Therapy for borderline personality disorder)
- Formal psychotherapy
- Group therapy

#### Types of outcome measures

We will examine a range of primary and secondary outcome measures. Other outcomes will be defined iteratively by the review team.

- *Primary outcome*: Suicidal ideation. Suicidal ideation is defined according to Beck’s ‘Scale for Suicide Ideation’ (SSI) [9] that measures *the intensity of current conscious suicidal intent,* examining *various dimensions of self-destructive thoughts or wishes*. We will not restrict the review a priori to specific measures of suicidal ideation.
- *Intermediate outcome*: Identification of suicide risk.
- *Secondary outcomes*: Suicide attempts, suicide, self-harm, hope, patient distress

### Information sources

Electronic databases will be searched from data of inception to present, and the search syntax will be modified as appropriate for use in the following databases: MEDLINE in Process (Ovid), PsycINFO (Ovid), EMBASE (Ovid), The Cochrane Central Register of Controlled Trials (CENTRAL) (Wiley Online Library) and CINHAL (EBSCO).

### Search strategy

A comprehensive search strategy has been developed, and we will use controlled vocabulary unique to each database (e.g. MEDLINE Medical Subject Headings (MeSH) terms) and free-term texts.

The strategy has been informed by discussions with experts in the field of psychiatry and systematic review methodology and by a prior scoping review identifying relevant keywords. An outline of the master search strategy for MEDLINE (OvidSP) has been developed and is presented in Table 1.

**Table 1:** Master search strategy

We used the following commands specific to the interface	
adj	Words have to appear next to each other.
$	Truncation symbol,
.ti,ab	Restricts the search to title and abstract fields
.tw	Restricts the search to title, keywords and abstract fields
EXP	Explode the subject heading, to retrieve more specific terms
/	MeSH heading.
?	Optional wild card character used within, or at the end of, a search term to substitute for one or no characters.
Once terms were compiled they then combined them using Boolean logic (AND, OR). We used The InterTASC Information Specialists' Sub-Group (ISSG)^3^ filter for Controlled studies (search 8 and 9 below).	
Master search developed in Ovid MEDLINE(R) in process and conducted on 16 June 2015 (6118 results)	
1	*suicide/ or exp suicidal ideation/ or exp suicide, attempted/
2	thoughts of death.ti,ab.
3	suicid*.ti,ab.
4	self-harm.ti,ab.
5	ending own life.ti,ab.
6	taking own life.ti,ab.
7	1 or 2 or 3 or 4 or 5 or 6
8	exp Clinical Trial/ or double-blind method/ or (clinical trial* or randomized controlled trial or multicenter study).pt. or exp Clinical Trials as Topic/ or ((randomi?ed adj7 trial*) or (controlled adj3 trial*) or (clinical adj2 trial*) or ((single or doubl* or tripl* or treb*) and (blind* or mask*))).ti,ab.
9	exp Case–control Studies/ or Control Groups/ or Matched-Pair Analysis/ or ((case* adj5 control*) or (case adj3 comparison*) or control group*).ti,ab.
10	8 or 9
11	exp Interpersonal Relations/ or exp Communication/ or communicat*.ti,ab.
12	(session* or appointment or meet* or encounter or explor* or clinical formulation or uncover* or disclos* or express* or inform* or mention* or reveal* or say* or speak* or probe or probing or dialogue or articulat* or contact).ti,ab.
13	(assess* or examin* or talk* or language or elicit* or evok* or ask* or dialogue or convers* or exchange or discus* or discourse or question* or interview* or consult* or interact* or counsel* or session* or appointment or meet* or encounter).ti,ab.
14	(online adj10 suicide).ti,ab.
15	(helpline adj10 suicide).ti,ab.
16	(treatment adj10 suicide).ti,ab.
17	(therapy adj10 suicide).ti,ab.
18	(admit or voice or verbalise or convey or clinical review or therapeutic alliance or relationship or doctor-patient relationship or support*).ti,ab.
19	risk assessment.ti,ab.
20	(verbal or non-verbal).ti,ab.
21	11 or 12 or 13 or 14 or 15 or 16 or 17 or 18 or 19 or 20
22	7 and 10 and 21

We will also use other methods used for identifying relevant research: We will search the ISRCTN registry (controlled trials register) and also perform hand searches of suicide journals, such as Suicide and Life-Threatening Behavior, The *Journal* of Crisis Intervention and *Suicide* Prevention, Archives of Suicide Research and Suicidology Online. We will also hand search reference lists of systematic reviews and included papers, and we will contact experts in this field; specifically, we will contact experts as defined by authors of included studies. Finally, we will perform reference checking and citation chasing (of included studies).

### Study records

#### Data management

EndNote X7.0.2 software will be used to manage references throughout the review. Once the searches have been run, results will be exported to EndNote and any duplicates automatically identified will be removed. This process will be assisted by hand searching for duplicates.

#### Screening

The review team

At least two reviewers will be involved at data extraction (overseen by the PenCLAHRC^2^ Evidence Synthesis Team). Two reviewers will screen titles and abstracts of studies identified through the searches, as well as in full paper screening and quality assessment, in order to minimise bias at all stages of the review.

Disagreement at any stage will be resolved through discussion and referral to a third reviewer. A PRISMA diagram will be completed to show the flow of the screening process and number of records at each stage.

Any conflicts of interest will be noted throughout at all stages. The review team has expertise in clinical communication, synthesising quantitative research and conducting systematic reviews.

#### Data extraction

All data will be extracted by one reviewer and checked by another. Data to be extracted and terminology will be defined in advance. For included studies, the primary reviewer will extract relevant information using the Cochrane Collaboration data extraction form for RCTs (Additional file 2). This will be adapted as appropriate for other study designs. The extraction form will be piloted before being finalised.

The assessment will be made by one reviewer and checked by a second.

#### Risk of bias

Risk of bias will be assessed using the Cochrane Collaboration’s tool for assessing risk of bias (Additional file 3). Two raters will independently assess the risk of bias for each study included in the full-text review.

#### Data synthesis

We will develop a PRISMA flow chart [7] of study selection based on the search strategy and inclusion/exclusion criteria. We will assess and analyse the included studies according to the Cochrane Handbook for Systematic Reviews of Interventions [9]. Where appropriate, we will use a random effects meta-analysis to combine study results. If this is not appropriate due to heterogeneity in study design, outcomes or participants, narrative synthesis will be undertaken. Our data synthesis will also differentiate between communication techniques that are used to elicit suicidal ideation and those used to respond to suicidal ideation.

#### Measures of treatment effect

We will report means or changes in mean scores (continuous data), and standardised mean differences (when different scales are used for the same outcome). The ratio of means method will be used to measure different outcomes. Binary outcomes and categorical data will be expressed as relative risks (RR) [10].

#### Dealing with missing data

When missing data are reported (i.e. methods, participants, statistics), we will contact the authors to request the missing data. We will contact them by email, using their email addresses on the publication or from the author’s documented affiliated organisation [9]. We will also describe the assumptions of any methods used for dealing with missing data.

#### Heterogeneity

We will assess differences between the included studies (differences in study design, types of participants, interventions, outcomes and intervention effects) [10] by applying the *I*^2^ statistic to meta-analytic results from each sub-group.

#### Sensitivity analysis

We will conduct sensitivity analysis both within and across different study designs as appropriate.

#### Data synthesis

We will conduct a meta-analysis only if participants, interventions and comparisons are sufficiently similar [10]. We will perform the meta-analysis using Stata data analysis and statistical software. If the included studies are too heterogeneous to conduct a meta-analysis, we will conduct a narrative synthesis [11].

#### Subgroup analysis

Anticipated subgroup analysis will be conducted for specific groups, e.g. sex, previous history of suicide attempts vs. new presentations, clinically different groups and robustness of the outcome measure.

#### Presentation of findings

We will assess each outcome by using the five GRADE considerations (study limitations, consistency of effect, imprecision, indirectness and publication bias) [10].

## Discussion

This systematic review will provide a detailed account of the existing evidence base for effective communication in eliciting and responding to suicidal thoughts.

Our interpretation of the synthesis of review findings will take account of limitations in studies identified and any limitations in our own review methodology. We will use multiple reviewers to help reduce time, as our first search (see Table 1) produced a large volume of studies to be screened and also to minimise the risk of bias.

The findings of this review will be of interest to healthcare professionals and paraprofessionals who are in contact with people potentially at risk of suicide. Evidence on effective communication techniques for eliciting suicidal thoughts and feelings will contribute to evidence-based suicide risk assessment. In addition, effective communication techniques for responding to suicidal thoughts and feelings will contribute to reducing patient distress arising from these thoughts and feelings. Both aspects will be used to inform training and practice of healthcare professionals and paraprofessionals in contact with people at risk of suicide.

## Endnotes

1. Responding within the therapeutic interaction

2. Collaboration for Leadership in Applied Health Research and Care, South West Peninsula

3. “Filters to Identify Randomized Controlled Trials and Other Trials”: University of Texas—school of public health https://sph.uth.edu/charting/Ovid_Medline_filters.htm (accessed 16.04.15).

## Additional files

Additional file 1:
**PRISMA-P 2015 Checklist: A checklist of recommended items to address in a systematic review protocol Cochrane Data extraction form for RCTs.** (DOC 85 kb) Additional file 2:
**Cochrane Data extraction form for RCTs: Data extraction form for interventions reviews of randomised controlled trials (RCTs).** (DOCX 73 kb) Additional file 3:
**Risk of bias assessment tool: A specific tool for assessing risk of bias in each included study.** (PDF 35 kb)

## Abbreviations

CINHAL: The Cumulative Index to Nursing and Allied Health Literature
ISRCTN: The International Standard Randomised Controlled Trial Number
MeSH: Medical Subject Headings
PenCLAHRC: Collaboration for Leadership in Applied Health Research and Care, South West Peninsula
RCT: randomised controlled trial
SSI: Scale for Suicide Ideation
