# Supplementary material for: Effective communication in eliciting and responding to suicidal thoughts: a systematic review protocol
Source: Syst Rev. 2016 Feb 17;5:31. doi: 10.1186/s13643-016-0211-y (PMC4758101; doi:10.1186/s13643-016-0211-y)
Supplement: Additional file 2: — Cochrane Data extraction form for RCTs: Data extraction form for interventions reviews of randomised controlled trials (RCTs). (DOCX 73 kb) [file 13643_2016_211_MOESM2_ESM.docx]

#
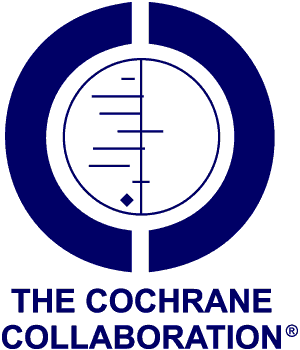
Data collection form

Intervention review – RCTs only

This form can be used as a guide for developing your own data extraction form. Sections can be expanded and added, and irrelevant sections can be removed. It is difficult to design a single form that meets the needs of all reviews, so it is important to consider carefully the information you need to collect, and design your form accordingly. Information included on this form should be comprehensive, and may be used in the text of your review, ‘Characteristics of included studies’ table, risk of bias assessment, and statistical analysis.

Notes on using a data extraction form:

- Be consistent in the order and style you use to describe the information for each report..
- Record any missing information as unclear or not described, to make it clear that the information was not found in the study report(s), not that you forgot to extract it.
- Include any instructions and decision rules on the data collection form, or in an accompanying document. It is important to practice using the form and give training to any other authors using the form.

| **Review title or ID** |
| --- |
|  |

| **Study ID** *(surname of first author and year first full report of study was published e.g. Smith 2001)* |
| --- |
|  |

| **Report IDs of other reports of this study** *(e.g. duplicate publications, follow-up studies)* |
| --- |
|  |

| **Notes:** |
| --- |

## General Information

| **Date form completed** *(dd/mm/yyyy)* |  |
| --- | --- |
| **Name/ID of person extracting data** |  |
| **Report title**  *(title of paper/ abstract/ report that data are extracted from)* |  |
| **Report ID**  *(ID for this paper/ abstract/ report)* |  |
| **Reference details** |  |
| **Report author contact details** |  |
| **Publication type**  *(e.g. full report, abstract, letter)* |  |
| **Study funding sources**  *(including role of funders)* |  |
| **Possible conflicts of interest**  *(for study authors)* |  |
| **Notes:** | |

## Study Eligibility

| **Study Characteristics** | **Eligibility sriteria**  *(Insert eligibility criteria for each characteristic as defined in the Protocol)* | | Yes | No | Unclear | **Location in text**  *(pg & ¶/fig/table)* |
| --- | --- | --- | --- | --- | --- | --- |
| **Type of study** | Randomised Controlled Trial | |  |  |  |  |
|  | Controlled Clinical Trial  *(quasi-randomised trial)* | |  |  |  |  |
| **Participants** |  | |  |  |  |  |
| **Types of intervention** |  | |  |  |  |  |
| **Types of outcome measures** |  | |  |  |  |  |
| **INCLUDE** | | **EXCLUDE** | | | | |
| **Reason for exclusion** |  | | | | | |
| **Notes:** | | | | | | |

DO NOT PROCEED IF STUDY EXCLUDED FROM REVIEW

## Population and setting

|  | **Description**  *Include comparative information for each group (i.e. intervention and controls) if available* | | **Location in text**  *(pg & ¶/fig/table)* |
| --- | --- | --- | --- |
| **Population description**  *(from which study participants are drawn)* |  | |  |
| **Setting**  *(including location and social context)* |  | |  |
| **Inclusion criteria** |  | |  |
| **Exclusion criteria** |  | |  |
| **Method/s of recruitment of participants** |  | |  |
| **Informed consent obtained** | Yes No Unclear |  |  |
| **Notes:** | | | |

## Methods

|  | **Descriptions as stated in report/paper** | | **Location in text**  *(pg & ¶/fig/table)* |
| --- | --- | --- | --- |
| **Aim of study** |  | |  |
| **Design** *(e.g. parallel, crossover, cluster)* |  | |  |
| **Unit of allocation**  *(by individuals, cluster/ groups or body parts)* |  | |  |
| **Start date** |  | |  |
| **End date** |  | |  |
| **Total study duration** |  | |  |
| **Ethical approval needed/ obtained for study** | Yes No Unclear |  |  |
| **Notes:** | | | |

## Risk of Bias assessment

*See* [*Chapter 8*](http://www.mrc-bsu.cam.ac.uk/cochrane/handbook/index.htm#chapter_8/8_assessing_risk_of_bias_in_included_studies.htm) *of the Cochrane Handbook*

| **Domain** | **Risk of bias** | | | **Support for judgement** | **Location in text**  *(pg & ¶/fig/table)* |
| --- | --- | --- | --- | --- | --- |
|  | Low risk | High risk | Unclear |  |  |
| **Random sequence generation**  *(selection bias)* |  |  |  |  |  |
| **Allocation concealment**  *(selection bias)* |  |  |  |  |  |
| **Blinding of participants and personnel**  *(performance bias)* |  |  |  | **Outcome group: All/** |  |
| *(if required)* |  |  |  | **Outcome group:** |  |
| **Blinding of outcome assessment**  *(detection bias)* |  |  |  | **Outcome group: All/** |  |
| *(if required)* |  |  |  | **Outcome group:** |  |
| **Incomplete outcome data**  *(attrition bias)* |  |  |  |  |  |
| **Selective outcome reporting?**  *(reporting bias)* |  |  |  |  |  |
| **Other bias** |  |  |  |  |  |
| **Notes:** | | | | | |

## Participants

*Provide overall data and, if available, comparative data for each intervention or comparison group.*

|  | **Description as stated in report/paper** | **Location in text**  (pg & ¶/fig/table) |
| --- | --- | --- |
| **Total no. randomised**  *(or total pop. at start of study for NRCTs)* |  |  |
| **Clusters**  *(if applicable, no., type, no. people per cluster)* |  |  |
| **Baseline imbalances** |  |  |
| **Withdrawals and exclusions**  *(if not provided below by outcome)* |  |  |
| **Age** |  |  |
| **Sex** |  |  |
| **Race/Ethnicity** |  |  |
| **Severity of illness** |  |  |
| **Co-morbidities** |  |  |
| **Other treatment received** *(additional to study intervention)* |  |  |
| **Other relevant sociodemographics** |  |  |
| **Subgroups measured** |  |  |
| **Subgroups reported** |  |  |
| **Notes:** | | |

## Intervention groups

*Copy and paste table for each intervention and comparison group*

**Intervention Group 1**

|  | **Description as stated in report/paper** | **Location in text**  *(pg & ¶/fig/table)* |
| --- | --- | --- |
| **Group name** |  |  |
| **No. randomised to group**  *(specify whether no. people or clusters)* |  |  |
| **Theoretical basis** *(include key references)* |  |  |
| **Description** *(include sufficient detail for replication, e.g. content, dose, components)* |  |  |
| **Duration of treatment period** |  |  |
| **Timing** *(e.g. frequency, duration of each episode)* |  |  |
| **Delivery** *(e.g. mechanism, medium, intensity, fidelity)* |  |  |
| **Providers**  *(e.g. no., profession, training, ethnicity etc. if relevant)* |  |  |
| **Co-interventions** |  |  |
| **Economic variables** *(i.e. intervention cost, changes in other costs as result of intervention)* |  |  |
| **Resource requirements to replicate intervention**  *(e.g. staff numbers, cold chain, equipment)* |  |  |
| **Notes:** | | |

## Outcomes

*Copy and paste table for each outcome.*

**Outcome 1**

|  | **Description as stated in report/paper** | | **Location in text**  *(pg & ¶/fig/table)* |
| --- | --- | --- | --- |
| **Outcome name** |  | |  |
| **Time points measured** |  | |  |
| **Time points reported** |  | |  |
| **Outcome definition** *(with diagnostic criteria if relevant)* |  | |  |
| **Person measuring/reporting** |  | |  |
| **Unit of measurement**  *(if relevant)* |  | |  |
| **Scales: upper and lower limits** *(indicate whether high or low score is good)* |  | |  |
| **Is outcome/tool validated?** | Yes No Unclear |  |  |
| **Imputation of missing data** *(e.g. assumptions made for ITT analysis)* |  | |  |
| **Assumed risk estimate**  (*e.g. baseline or population risk noted in Background)* |  | |  |
| **Power** |  | |  |
| **Notes:** | | | |

## Results

*Copy and paste the appropriate table for each outcome, including additional tables for each time point and subgroup as required.*

***Dichotomous outcome***

|  | **Description as stated in report/paper** | | | | | **Location in text**  *(pg & ¶/fig/table)* |
| --- | --- | --- | --- | --- | --- | --- |
| **Comparison** |  | | | | |  |
| **Outcome** |  | | | | |  |
| **Subgroup** |  | | | | |  |
| **Timepoint** *(specify whether from start or end of intervention)* |  | | | | |  |
| **Results** | **Intervention** | | | **Comparison** | |  |
|  | No. events | No. participants | | No. events | No. participants |  |
|  |  |  | |  |  |  |
| **No. missing participants and reasons** |  | | |  | |  |
| **No. participants moved from other group and reasons** |  | | |  | |  |
| **Any other results reported** |  | | | | |  |
| **Unit of analysis** *(by individuals, cluster/groups or body parts)* |  | | | | |  |
| **Statistical methods used and appropriateness of these methods** *(e.g. adjustment for correlation)* |  | | | | |  |
| **Reanalysis required?** *(specify)* | Yes No Unclear | |  | | |  |
| **Reanalysis possible?** | Yes No Unclear | |  | | |  |
| **Reanalysed results** |  | | | | |  |
| **Notes:** | | | | | | |

***Continuous outcome***

|  | | **Description as stated in report/paper** | | | | | | | **Location in text**  *(pg & ¶/fig/table)* | |
| --- | --- | --- | --- | --- | --- | --- | --- | --- | --- | --- |
| **Comparison** | |  | | | | | | |  | |
| **Outcome** | |  | | | | | | |  | |
| **Subgroup** | |  | | | | | | |  | |
| **Timepoint** *(specify whether from start or end of intervention)* | |  | | | | | | |  | |
| **Post-intervention or change from baseline?** | |  | | | | | | |  | |
| **Results** | **Intervention** | | | | | **Comparison** | | |  | |
|  | Mean | | SD (or other variance) | No. participants | | Mean | SD (or other variance) | No. participants |  | |
|  |  | |  |  | |  |  |  |  |  |
| **No. missing participants and reasons** | |  | | | |  | | |  | |
| **No. participants moved from other group and reasons** | |  | | | |  | | |  | |
| **Any other results reported** | |  | | | | | | |  | |
| **Unit of analysis**  *(individuals, cluster/ groups or body parts)* | |  | | | | | | |  | |
| **Statistical methods used and appropriateness of these methods** *(e.g. adjustment for correlation)* | |  | | | | | | |  | |
| **Reanalysis required?** *(specify)* | | Yes No Unclear | | |  | | | |  | |
| **Reanalysis possible?** | | Yes No Unclear | | |  | | | |  | |
| **Reanalysed results** | |  | | | | | | |  | |
| **Notes:** | | | | | | | | | |  |

***Other outcome***

|  | **Description as stated in report/paper** | | | | | **Location in text**  *(pg & ¶/fig/table)* |
| --- | --- | --- | --- | --- | --- | --- |
| **Comparison** |  | | | | |  |
| **Outcome** |  | | | | |  |
| **Subgroup** |  | | | | |  |
| **Timepoint** *(specify whether from start or end of intervention)* |  | | | | |  |
| **Results** | Intervention result | SD (or other variance) | | Control result | SD (or other variance) |  |
|  |  |  | |  |  |  |
|  | Overall results | | | SE (or other variance) | |  |
|  |  | | |  | |  |
| **No. participants** | Intervention | | | Control | |  |
|  |  | | |  | |  |
| **No. missing participants and reasons** |  | | |  | |  |
| **No. participants moved from other group and reasons** |  | | |  | |  |
| **Any other results reported** |  | | | | |  |
| **Unit of analysis** *(by individuals, cluster/groups or body parts)* |  | | | | |  |
| **Statistical methods used and appropriateness of these methods** |  | | | | |  |
| **Reanalysis required?** *(specify)* | Yes No Unclear | |  | | |  |
| **Reanalysis possible?** | Yes No Unclear | |  | | |  |
| **Reanalysed results** |  | | | | |  |
| **Notes:** | | | | | | |

## Applicability

| **Have important populations been excluded from the study?** *(consider disadvantaged populations, and possible differences in the intervention effect)* | Yes No Unclear |  |
| --- | --- | --- |
| **Is the intervention likely to be aimed at disadvantaged groups?** *(e.g .lower socioeconomic groups)* | Yes No Unclear |  |
| **Does the study directly address the review question?**  *(any issues of partial or indirect applicability)* | Yes No Unclear |  |
| **Notes:** | | |

## Other information

|  | **Description as stated in report/paper** | **Location in text**  *(pg & ¶/fig/table)* |
| --- | --- | --- |
| **Key conclusions of study authors** |  |  |
| **References to other relevant studies** |  |  |
| **Correspondence required for further study information** *(from whom, what and when)* |  | |
| **Notes:** | | |
